# Surface Tailoring of MoS_2_ Nanosheets with Substituted Aromatic Diazonium Salts for Gas Sensing: A DFT Study

**DOI:** 10.1021/acsomega.4c04506

**Published:** 2024-08-23

**Authors:** Rabiaa Hajlaoui, Sabrine Baachaoui, Sami Ben Aoun, Said Ridene, Noureddine Raouafi

**Affiliations:** † Advanced Materials and Quantum Phenomena Laboratory, Department of Physics, Faculty of Sciences of Tunis, Tunis El Manar University, Tunis 2092, Tunisia; ‡ Analytical Chemistry and Electrochemistry Lab (LR99ES15), Department of Chemistry, Faculty of Sciences, University of Tunis El Manar, Tunis 2092, Tunisia; § Department of Chemistry, Faculty of Science, Taibah University, Al-Madinah Al-Munawwarah 30002, Saudi Arabia

## Abstract

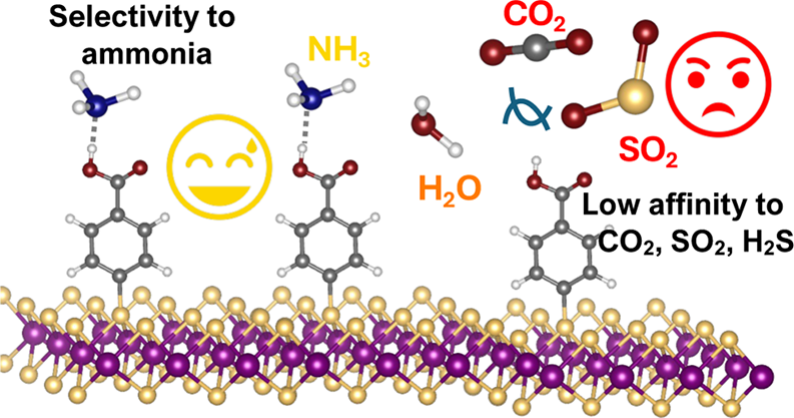

Two-dimensional (2D) nanomaterials are useful for building gas sensors owing to their desirable electronic and optical properties. However, they usually suffer from selectivity, because they cannot discriminate between gas molecules. Functionalization with organic molecules can be used to tailor their surfaces to recognize a specific family of compounds. In this study, solid-state density functional theory (DFT) was used to elucidate the functionalization of MoS_2_ with substituted aromatic diazonium salts (*R* = −H, – CH_3_, −CO_2_H, −CHO, −OCH_3_, and −NO_2_). Results showed that chemical reaction with diazonium salts is favored to their physical adsorption (*E*_ads_ = −0.04 to −0.38 eV vs *E*_rxn_ = −1.47 to −2.20 eV), where organic cations have a preference to attach atop of sulfur atoms. Chemical functionalization induced a small variation in the bandgap energy not exceeding 0.04 eV; thus, the optical properties were well preserved. In the presence of ammonia, the substituted **MoS**_**2**_**/2(a–f)** responded to the target analyte through a change in the interaction energy, varying from −0.08 to −0.83 eV, where the best interaction energy was obtained for **MoS**_**2**_**/2c**, bearing the carboxylic acid group. In the presence of other gases such as CO_2_, SO_2_, and H_2_S, the interaction energy is lower (−0.14 to −0.35 eV), indicating good selectivity of the nanomaterials. Furthermore, the interaction increased in the presence of humidity, which was more realistic than that in the presence of neat NH_3_. This interaction was confirmed by computing the partial charges. Recovery times estimated from the interaction energies ranged from 0.31 s to several minutes, depending on the interacting molecules. Phenylcarboxyl-modified MoS_2_ nanosheets show great potential as candidates for the development of chemoresistive gas sensors that are specifically designed for detecting ammonia.

## Introduction

In the past decade, the domain of 2D materials has experienced rapid growth, presenting exciting prospects for electronic device manufacturing, gas sensing, and biosensing applications.^1−2^ Despite the significant attention garnered by graphene owing to its remarkable properties,^1,4−7^ other 2D materials, such as transition metal dichalcogenides (TMDCs) like MoS_2_, have emerged as compelling research subjects because of their distinctive physical and chemical characteristics. Exploration of bandgap engineering in 2D materials,^2,8^ including MoS_2_, have involved studying different layer configurations (monolayer, bilayer, and bulk), each exhibiting distinct physicochemical attributes.^9−11^ For instance, MoS_2_ demonstrates a bandgap transition from direct to indirect as the number of layers increases.^12^

Functionalizing the surfaces of these materials by charge-transfer doping *via* physisorption or covalent bonding through chemisorption allows access to vast chemical spaces. Surface modification of metallic 2D TMDCs by grafting organic functional groups has been extensively researched and proven to be effective in modulating their properties. However, this technique has only been successfully demonstrated on metallic 2D TMDCs, such as the 1T phase of MoS_2_.^3^ Recent studies have focused on covalent functionalization, specifically on the 2H phase of MoS_2_.^13^ Covalent functionalization, particularly *via* bonds formed between functional groups and surface sulfur (Se or Te in other TMDCs), plays a crucial role in gas sensing applications, allowing tailored responses to different gases. Additionally, functionalization facilitates the meticulous management of surface chemistry and morphology, thereby amplifying gas-sensing capabilities and potentially finding utility in environmental monitoring, industrial safety, and the healthcare sector. Recently, it was shown that the functionalization of MoS_2_ with certain functional groups, such as amine or thiol groups, can tailor its response to different gases.^14,15^ Aryl diazonium salts, featuring an R–aryl–N_2_^+^ structure, are frequently used as surface modifiers because of their convenient functionalization and characterization methods.^16−19^ The versatility of these salts, with their ability to substitute various functional groups depending on the application, render them a valuable tool for tailoring material surfaces in sensors, cathodes, catalysts, and other applications.^16,20,21^ Functionalization of MoS_2_ with aryl diazonium salts has been shown to enhance their properties, offering a means to customize both chemical and electronic characteristics.^22^ This process is now widely recognized as a prominent covalent functionalization pathway for various nanomaterials, generating highly reactive aromatic cations upon the release of N_2_ molecules.

Atmospheric pollution is a major environmental concern. The development of highly sensitive and selective toxic gas sensors is important goal to be achieved. In this direction, 2D-TMDs are promising materials for use in gas sensors owing to their high surface-to-volume ratio. Numerous applications such as pollutant detection, industrial safety, and air quality monitoring depend on gas detection. MoS_2_ and other semiconductor materials have attracted considerable interest because of their unique qualities and potential for use in high-sensitivity gas sensors. Nonetheless, enhancing the stability and selectivity of these sensors remains a significant issue. Pure MoS_2_ has been widely employed to detect many gases in the atmosphere, including NO_2_,^23^ NO,^24^ and NH_3_,^25^ based on the previous theoretical results. The researchers employed a method of doping with metals and other functionalizations to further alter the system characteristics to boost its interaction with gas molecules, because pristine MoS_2_ did not interact very well with gas molecules. For instance, Zhu et al. studied the adsorption properties of common gases (CO, NO_2_, H_2_O, and NH_3_) on a pristine MoS_2_ monolayer and on a metal-doped MoS_2_ monolayer (V, Nb, and Ta),^26^ showing that metal doping can significantly improve the adsorption properties, chemical activity, and sensitivity of MoS_2_ monolayer. Ma *et al.* improved the detection capabilities of MoS_2_ for CO and NO by doping the MoS_2_ monolayer with other metals (Au, Pt, Pd, and Ni).^27^ The results suggested that the addition of appropriate dopants could be an effective method for improving the performance of MoS_2_-based gas sensors. Late *et al.* developed a chemical sensor to detect gases such as NO_2_, NH_3_, and H_2_ using a MoS_2_-based FET formed by a single to multiple layers of MoS_2_ deposed on SiO_2_/Si substrates.^28^ These sensors can easily detect analytes such as NH_3_ and NO_2_ at concentrations below 1 ppm.^29^

MoS_2_, in addition to its functionality in gas sensing, has also demonstrated potential in the fields of environmental remediation and energy storage devices. Studies have shown that the combination of MoS_2_ with other nanomaterials, such as CdS and TiO_2_ nanotubes, results in enhanced photoelectrochemical properties.^30^ Moreover, rGO-MoS_2_ heterostructures have been utilized for the efficient removal of hazardous organic pollutants and ofloxacin from water bodies.^31,32^ Additionally, mechanically exfoliated MoS_2_ nanosheets, when combined with conductive polyaniline or in the form of MoS_2_-*g*-C_3_N_4_ heterostructures, have been employed in the construction of high-performance supercapacitor electrodes.^33,34^

In this study, we examined the band structure of covalently modified 1H-MoS_2_ monolayers using several electron-withdrawing and electron-donating aromatic diazonium salts via DFT calculations. We examined the physical and chemical alterations in pristine MoS_2_ through the introduction of aryl diazonium salts, resulting in chemically modified MoS_2_, focusing on six modifiers to evaluate the reaction energy and its dependence on the added functional groups. Furthermore, we assessed the adsorption and sensing properties of modified MoS_2_ monolayers, proposed stable adsorption structures for different gases, and elucidated their thermodynamic properties. Our study provides insights into the gas-sensing response of 4-substituted phenyl-functionalized MoS_2_ nanomaterial-based sensors and offers valuable implications for sensor design and development.

## Computational Details

### Simulation Parameters

Theoretical computations were conducted using first-principles employing DFT within the Quantum ESPRESSO package v7.0.^35^ The Perdew–Burke–Ernzerhof (PBE) version of the generalized gradient approximation characterized the exchange-correlation density functional.^36^ Vanderbilt ultrasoft pseudopotentials were used to model the interaction between the valence electron and the ionic cores.^37^ A 50-Ry energy cut off was employed for the plane-wave basis set with a charge density set at 450 Ry. The force convergence threshold for geometry optimization was set to 1 × 10^–3^ Ry/Bohr, and the electronic convergence threshold was set to 1 × 10^–6^ Ry. For all the single-point calculations, this threshold was lowered to 1 × 10 ^–9^ Ry.

To simulate the functionalization process of the 2D MoS_2_ nanosheets, only one side of the 4 × 4-supercell of the 1H-MoS_2_ monolayer was considered for chemical modification using reactive intermediates. This material possesses a hexagonal crystal structure space group (P63/mmc), comprising 16 Mo and 32 S atoms.

A 4 × 4 × 1 *k*-point mesh was utilized for Brillouin zone sampling during geometric optimization of the metal atoms on the MoS_2_ monolayer. Additionally, an 8 × 8 × 1 *k*-point grid ensured the convergence of the density of states. A 20 Å vacuum was applied along the *z*-axis perpendicular to the 2D sheet to prevent interactions between the periodic images. The lateral dimensions of the simulation box are 12.76 Å × 12.76 Å.

### Adsorption Sites

**Figure 1:**
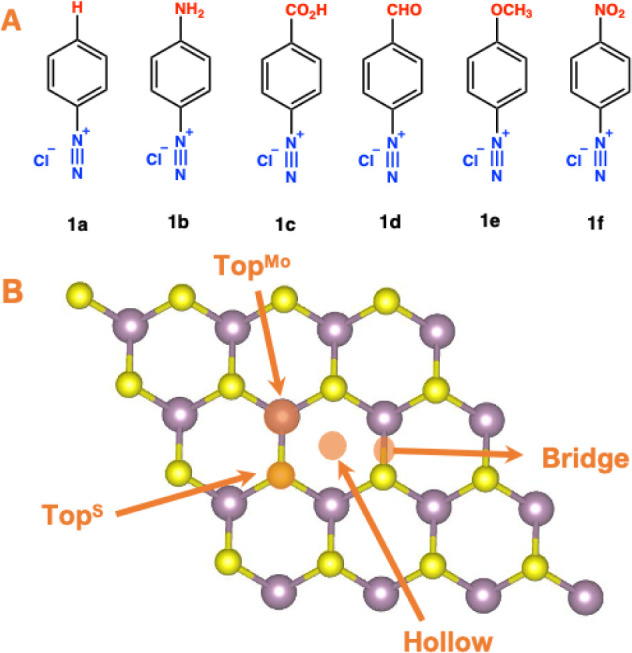
(A) Chemical structures of various diazonium salts examined for surface alteration of MoS_2_ nanosheets; (B) Depiction of the MoS_2_ nanosheet within a 4 × 4 supercell. The adsorption sites: Top^Mo^, Top^S^, bridge, and hollow are highlighted by the arrows. Mo and S are denoted by the purple and yellow spheres, respectively.

To identify the most stable adsorption sites with high adsorption energies for organic molecules on the MoS_2_ surface, we constructed configurations in which the molecules were positioned on specific high-symmetry adsorption sites. These sites included top^Mo^, top^S^, bridges, and hollows, as illustrated in Figure 1.

### Calculation of the Interaction Energies

The interaction strength between the pristine MoS_2_ and the adsorbates was assessed by calculating the adsorption energies. The adsorption energy of aryldiazonium, Δ*E*_ads_, was calculated using eq 1):
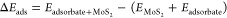
1where  is the energy of the system, and  and *E*_adsorbate_ are the energies of the monolayer MoS_2_ and adsorbate, respectively. A negative value of *ΔE*_ads_ indicates favorable adsorption. A similar treatment was used to determine the reaction energies between pristine MoS_2_ and the reactive intermediates.

The reaction of the sulfur atoms as nucleophiles with the R-Aryl^+^ cation induced surface deformation by weakening the Mo–S bonds. Recent studies have shown that the deformation energy could be substantial in the case of the functionalization of graphene or BC_6_N with reactive intermediates.^6^ The deformation energy, *ΔE*_def_, of the MoS_2_ nanosheet following the adsorption of diazonium salts was estimated using eq 2):
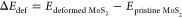
2where  and  are the energies of deformed and pristine MoS_2_, respectively.

The interaction energy values of the functionalized MoS_2_ (complex) with the adsorbates, *ΔE*_int_, were determined from the difference in the complex (modified MoS_2_ + adsorbate) energy, *E*_complex_, modified MoS_2_ energy , and adsorbate energy *E*_adsorbate_ using eq 3):
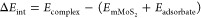
3

The interaction energy between the modified surface and ammonia in the presence of water molecules was also estimated. We computed the overall interaction energy and later subtracted the interaction energy of water–ammonia determined from single-point calculations of the stable complex (eq 4).
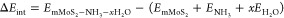
4

With *x* = 1 or 2

### Calculation of the Recovery Times and the Charge Density Differences

According to the Van’t Hoff–Arrhenius theory, the recovery time, denoted as τ, can be determined using eq 5):^38^
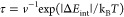
5where *T* is the operating temperature, k_B_ is Boltzmann’s constant, and Δ*E*_int_ is the interaction energy. The attempt frequency, ν, was assumed to be 1 × 10^12^ Hz.

The charge density differences, *Δρ*, were derived from the individual charge densities of the fully relaxed minimal-energy systems of the complex (ρ_complex_), modified MoS_2_ (), and target (ρ_target_) using eq 6):
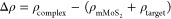
6

Following the acquisition of charge density difference profiles, we processed to quantify the impact of covalent bonding through Löwdin population analysis.^39^

Additionally, our attention was directed toward acquiring comprehensive electronic structures of functionalized 1H-MoS_2_ This was achieved by generating a band diagram, wherein spatial information for electronic states was scrutinized through the plotting of partial charge densities using the Vesta program.^40^

## Results

### Energetics of the Adsorption of Diazonium Salts

Figure 1A shows the six diazonium salts investigated in this study. They differed according to the radical at the para-position of the diazonium group. The reason for this choice was to evaluate the effect of substituting the hydrogen atom in **1a** with electron-donating (−NH_2_ and −OCH_3_) or electron-withdrawing groups, such as −CO_2_H, −CHO, and −NO_2_ in **1b**–**1f**.

### Physical Adsorption

Commencing with the fully relaxed, minimum energy configurations of both the adsorbates and the MoS_2_ supercell, the organic functionalizers were situated 3.5 Å above the surface of 1H-MoS_2_, and the chlorine atoms in all geometries were positioned at a distance of 2.0 Å from the surface to ensure the overall neutrality of the supercell.

Several possible positions for the starting adsorbates were investigated (Figure 1B). The examined positions are atop the Mo or S atoms, bridging the Mo–S bond or on the hollow position in the hexagon formed by three sulfur and three molybdenum atoms. The interaction energies for the physical adsorption of diazonium salts (**1a**–**1f)** on the MoS_2_ surface are listed in Table 1. Analyses of the data reveals a spectrum of adsorption energies spanning from −0.03 to −0.38 eV, furthermore it is evident that there is a consistent energetic preference for the top^Mo^ adsorption sites across all adsorbates from **(1b)** to **(1f)** but in case of **1a**, it is slightly favored for the top^S^ position.

**Table 1:** Physical and Chemical Adsorption Energies and the Distances Separating Adsorbates **1a**–**1f** and the MoS_2_ Surface and for the Adducts **MoS**_**2**_**/(2a**–**2f)**

systems	*E*_ads_ /eV	*d*_S–N_ /Å	*E*_def_ /×10^–3^ eV
**MoS_2_/1a**	–0.24^a^	3.46	4
	–0.27^b^	3.29	6
	–0.26^c^	3.37	7
	–0.04^d^	4.37	0.1
**MoS_2_/1b**	–0.38^a^	3.40	6
	–0.38^b^	3.34	8
	–0.36^c^	3.20	10
	–0.07^d^	3.85	0.2
**MoS_2_/1c**	–0.26^a^	3.45	4
	–0.27^b^	3.50	5
	–0.25^c^	3.56	3
	–0.04^d^	3.76	0.1
**MoS_2_/1d**	–0.26^a^	3.66	5
	–0.27^b^	3.49	4
	–0.23^c^	3.30	5
	–0.03^d^	3.87	0.1
**MoS_2_/1e**	–0.32^a^	3.37	6
	–0.33^b^	3.33	7
	–0.31^c^	3.20	8
	–0.06^d^	4.05	2
**MoS_2_/1f**	–0.28^a^	3.61	4
	–0.29^b^	3.56	5
	–0.27^c^	3.50	4
	–0.03^d^	3.91	0.2
**MoS_2_/2a**	–1.65	1.81^e^	110
**MoS_2_/2b**	–2.20	1.77^e^	70
**MoS_2_/2c**	–1.62	1.81^e^	100
**MoS_2_/2d**	–1.54	1.81^e^	110
**MoS_2_/2e**	–1.99	1.79^e^	90
**MoS_2_/2f**	–1.47	1.81^e^	110

a Atop position of S.

b Atop position of Mo.

c Bridge position.

d Hollow position.

e S–C covalent bond length.

Geometrically, it was evident that all diazonium cations were positioned at shorter distances from the Mo atom than from the S atom. The N···S distance ranges from 3.37 to 3.66 Å, while the N···Mo distance spans from to 3.29 to 3.56 Å. The optimized geometries of the top^Mo^ sites for all the pristine MoS_2_ sheets/adsorbates are shown in Figure S1.

Following the physical adsorption of diazonium salts, the MoS_2_ nanosheet experienced a slight deformation owing to the charge repulsion of the nitrogen lone pair with the electronic cloud of molybdenum disulfide. As presented in Table 1, the calculated deformation energies are equal to or less than 0.1 eV, indicating the weak effect of the adsorbate on the electronic cloud of MoS_2_ because of the large distance separating them (∼3.4 to ∼3.7 Å).

### Chemical Reaction

**Figure 2:**
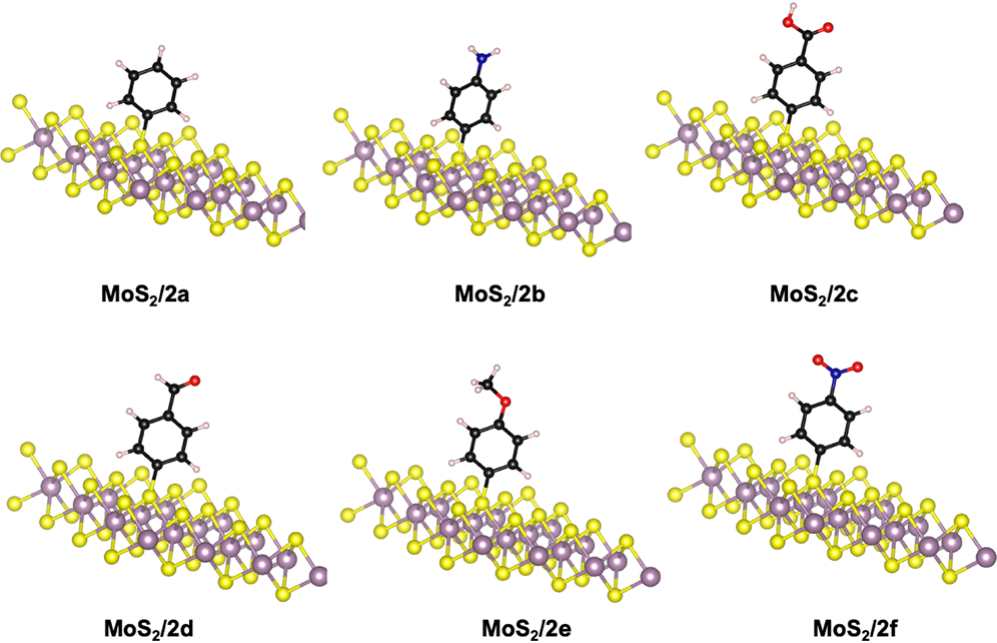
Optimized geometries of the chemical reaction adducts of the MoS_2_ surface functionalized with a) benzene, b) aminobenzene, c) carboxybenzene, d) formylbenzene, e) methoxybenzene, and f) nitrobenzene groups.

The aryl cations obtained from the splitting of diazonium salts underwent a reaction with a single bond from MoS_2_, resulting in the formation of 4-substituted phenyl-functionalized-MoS_2_ adducts and the release of dinitrogen molecules. The S-aniline, in MoS_2_/2b, bond exhibits the shortest length among all the groups studied (1.77 Å), whereas the other S–C(phenyl) covalent bond lengths are 1.79 to 1.81 Å and, similar to other heterocycles, have no experimental comparison (Figure 2).

In the case of chemical reaction, the energy values are more sizable compared to the physical adsorption of diazonium salts, and ranges from −1.47 to −2.20 eV. These values are 3.8- to 5.8-fold higher than those of the physical interaction energies, indicating that the chemical reaction is favored over chemical adsorption of the functionalizers. Furthermore, considering the unsubstituted benzene ring (**2a**) as a reference adsorbate, the electron donating groups in **2b** and **2e** made the adsorption more difficult since it needs more −0.34 to −0.55 eV to take place compared to the electron withdrawing groups in **2c**, **2d**, and **2f** which in the contrary help it. Indeed, the energy is lower by 0.03 to 0.18 eV compared to that obtained with **2a**. The most significant effect was observed for the nitro group (**2f**), which had the highest electron-withdrawing effect. This can be explained by the opposing electronic effects that stabilize or destabilize the formed aromatic cation, making it more or less prone to reacting with the sulfur atom from MoS_2_.

A comparison between the deformation energies for the chemical reaction and physical adsorption showed that the former is more sizable, ranges from 0.07 to 0.11 eV. The lower values of strain energy for the physical adsorption of diazonium salts on MoS_2_ compared with their chemical reaction can be attributed to the deformation in the S–C interaction zone and the sharing of lone pairs of electrons with the 4-substituted phenyl cations.

### Band Structure and Density of States

#### Band Structure

Band structure analysis revealed that pristine MoS_2_ has a direct bandgap of approximately 1.6 eV, which is characteristic of a monolayer of transition metal dichalcogenides (Figure S2).^41,42^ This attribute makes it suitable for optoelectronic and electronic applications, such as transistors and photodetectors. Both the valence band maximum (VBM) and conduction band minimum (CBM) are located at the K point in the Brillouin zone. The band structure exhibited symmetry around the Fermi level (*E*_f_), which was set as zero in the plot. Although the effective masses of electrons and holes in MoS_2_ are relatively high, leading to low charge carrier mobility, the bandgap can be tailored by applying strain or surface functionalization with various adsorbates. This modification can alter the electronic properties of MoS_2_ and tune its band gap.

**Figure 3:**
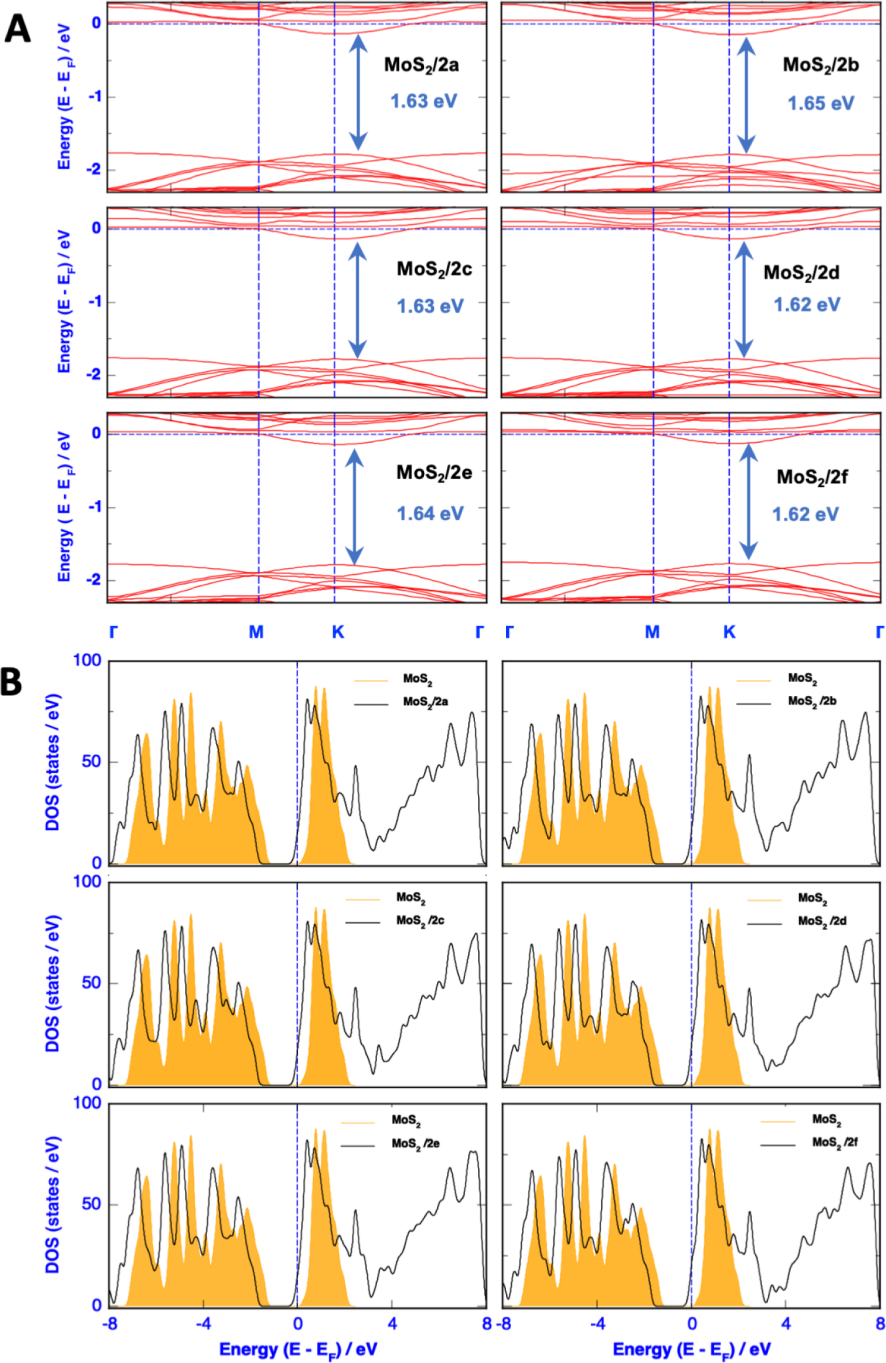
(A) Band structures and (B) total density of states (DOS) of modified **MoS_2_/2a-–f**. The filled orange areas in (B) indicate the total DOS of the pristine MoS_2_ for comparison.

The bandgap of the functionalized MoS_2_ with the six aryl 4-substituted aryl cations was calculated, as shown in Figure 3A. Compared to pristine MoS_2_, the bandgap of the modified MoS_2_ nanosheets (**MoS**_**2**_**/2a–MoS**_**2**_**/2f**) varied from 0.01 to 0.04 eV. A decrease in the bandgap represents an improvement in the conductivity; however, its magnitude is weak. This suggests that the transition of electrons from the valence band to the conduction band occurs readily, thereby enhancing the conductivity of the system.

#### Total DOS

All DOS plots show the presence of new peaks (from ∼2 to ∼8 eV) above the Fermi level because of the electronic clouds of the newly introduced organic functionalizers (Figure 3B). Furthermore, the Fermi level shifted toward the VBM, and the modified MoS_2_ nanosheets became p-doped and probably slightly conductive compared with the semiconducting pristine MoS_2_. This was probably due to the introduction of a positive charge by the aryl cations, which was widespread over the entire supercell. The Fermi level shift was larger with the 4-nitrophenyl group than with the 4-aminophenyl group because of the opposite electronic effect of the para-substituted groups.

#### Projected DOS

**Figure 4:**
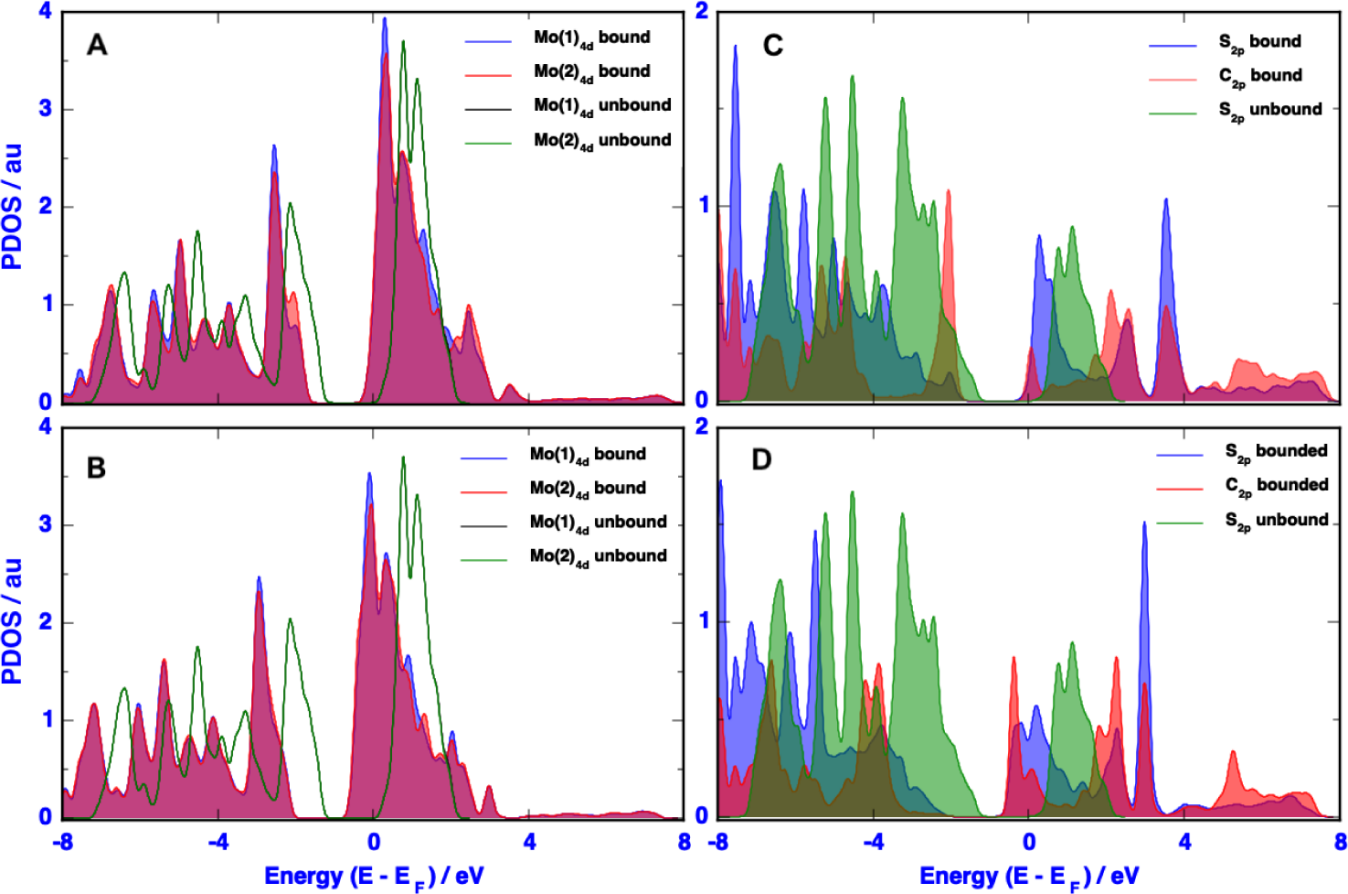
PDOS of Mo (4d), S(2p), and C(2p) orbitals from pristine MoS_2_ and (A and B) functionalized MoS_2_/2b and(C and D) MoS_2_/2f nanosheets.

The projected DOS (PDOS) for the two molybdenum atoms and the bridging sulfur atom, which is the side of the chemical reaction, was plotted before and after chemical bonding of the para-substituted phenyl groups with the sulfur atom (Figure 4). Because of the symmetry effect, the PDOS plots of the two unbound Mo atoms were superimposed (black and green lines in panels **4A** and **4 B**, respectively). After the reaction, the energies of the peaks of the Mo(4d) orbitals shifted to lower values owing to their binding to the carbocation. Panels 4C and 4D show the S(2p) orbital plots before and after binding to the carbocation from the aryl groups. In addition, the orbital peaks changed drastically after chemical bonding with the carbon atom (compare the green and blue plots). The red plot corresponds to the carbocation atom introduced by the para-substituted aryl groups.

Effect of para-substituent groups on the phenyl ring (panels **4A** and **4B**) on the 4-aminophenyl ring (**panel 4A**) and 4-nitrophenyl ring (**panel 4 B**). The latter induces a more pronounced effect on the Mo(4) orbital peaks. Indeed, one can notice a larger shift of ∼1.0 eV peak after functionalizing the surface with the 4-nitrophenyl group compared to the 4-aminophenyl ring (shifted by only ∼0.5 eV). Similarly, the S(2p) orbital peaks split and shifted concomitantly to higher and lower energy values. The two peaks that initially appear at ca. 1.15 eV split into two peaks appearing at ∼0.3 and ∼3.5 eV (**panel 4A**), and ∼0.2 and ∼3.0 eV (**panel 4B**). The C(2p) orbital peaks (red filled areas) in the same two panels display quite different peaks at different energy positions because of the opposite electronic effects of the 4-amino and 4-nitro substituent groups.

### Gas Sensing

#### Thermodynamics

To investigate the gas-detection performance of the aryl-functionalized MoS_2_ monolayers, we studied the thermodynamic properties of these surfaces in the presence of ammonia gas, a typical pollutant gas indicative of industrial pollution or medical conditions.^43,44^ The results summarized in Table 2 illustrate that the functional groups engaged with the N–H or O–H groups of the amine or acid through hydrogen bonding. The lengths of these hydrogen bonds span from 1.46 to 2.54 Å. All the relaxed geometries interacting with NH_3_ are shown in Figure S3. Furthermore, as indicated in Table 2, the interaction energies, with a single molecule of NH_3_, vary in a wide range, from −0.08 to −0.68 eV for the N–H/O and O–H/N bonds, respectively, between the ammonia N–H (carboxylic acid O–H) and the functional groups. Notably, ammonia strongly interacted with the carboxylic acid group of benzoic acid on the **MoS**_**2**_**/2c** substrate. The weakest hydrogen bonding is observed for the **MoS**_**2**_**/2f** substrate in the NO_2_ group. The strongest hydrogen bonds (HBs) are the shortest Y–H···X (X and Y = N and O) bonds and vice versa. In particular, the strongest interaction between – CO_2_H···N–H resulted from two asymmetric HBs between O–H···N (1.46 Å) and N–H···C = O (2.66 Å).

**Table 2:** Gas Adsorption on the Functionalized MoS_2_ Nanosheets, their Interaction Energies, the Lengths of Established Hydrogen Bonds, and the Recovery Times Calculated for the Adsorption of NH_3_, H_2_O, 2 × NH_3_, NH_3_/H_2_O, and NH_3_/2 × H_2_O on the Surface of **MoS**_**2**_**/2c** Nanosheets at 298 and 373 K

targets	substrates	*E*_inter_/eV	HB lengths/*A*°	τ (298 K)/s	τ (373 K)/s
NH_3_	**MoS_2_/2a**	–0.12	C–H···N = 2.53	0.31	0.001
	**MoS_2_/2b**	–0.31	N–H···N = 2.07		
	**MoS_2_/2c**	–0.68	O–H···N = 1.62		
			C==O···H= 2.66		
	**MoS_2_/2d**	–0.08	C≡O···H = 2.27		
		–0.14	C–H···N = 2.54		
	**MoS_2_/2e**	–0.12	C–H···N = 2.52		
	**MoS_2_/2f**	–0.08	N==O···H = 2.42		
CO_2_	**MoS_2_/2c**	–0.15	O–H···O = 1.99		
SO_2_	**MoS_2_/2c**	–0.34	O–H···O = 1.79		
H_2_S	**MoS_2_/2c**	–0.27	C=O···H = 2.30		
			O–H···S = 2.30		
H_2_O	**MoS_2_/2c**	–0.54	C=O···H = 1.88	0.001	<0.001
			O–H···O = 1.69		
2 × NH_3_	**MoS_2_/2c**	–0.83 (−1.15)^a^	O–H···N = 1.46	108.73	0.163
			N–H···N = 1.96		
			C==O···H = 1.92		
NH_3_/H_2_O	**MoS_2_/2c**	–0.94 (−1.35)^a^	O–H···N = 1.49	7881.88	5.019
			C=O···H = 1.76		
			N–H···O = 1.90		
NH_3_/2 × H_2_O	**MoS_2_/2c**	–0.75 (−1.04)^a^	O–H···N = 1.55	4.82	0.0135
			N–H···O = 2.08		
			O–H···O = 1.89		

a Values in parentheses represent the sum of all HB energies.

Furthermore, the adsorption of two NH_3_ molecules was studied to account for the effect of ammonia concentration on the interaction energy. The results showed the latter is higher when two NH_3_ are adsorbed compared to that obtained for one adsorbate (−0.83 eV vs −0.68 eV) and the O–H···N distance that is ∼10% shorter (Table 2). Indeed, the carboxylic acid group formed two hydrogen bonds (O–H···N and C=O···H) with the NH_3_ molecules, and the two NH_3_ molecules formed a third N–H···N hydrogen bond (Figure S5 A). The cooperative HB dense network resulted in a higher interaction energy between the target species and the sensing surface.

#### Selectivity

Covalent functionalization of MoS_2_ aims to develop selective MoS_2_-based sensors for the detection of specific gases, such as CO_2_, SO_2_, H_2_S, and H_2_O, for medical diagnosis or environmental pollution monitoring.^43,44^ These four molecules were examined with the CO_2_H group from **MoS**_**2**_**/2c** as potential interfering gases. All relaxed geometries at the energy minima are plotted in Figure S4. As shown in Table 2, the interaction energies exhibit a broad spectrum, ranging from −0.15 to −0.54 eV for the O–H···O, C = O···H, and O–H···S hydrogen bonds formed between the carboxylic acid and the various gases. The bond lengths range from 1.69 to 2.30 Å, depending on the strength of the interaction. The strongest interaction was observed with H_2_O. Since the adsorption energies of NH_3_ and H_2_O adsorbates are −0.68 eV and −0.54 eV, respectively, this makes the surface potentially not selective to ammonia.

**Figure 5:**
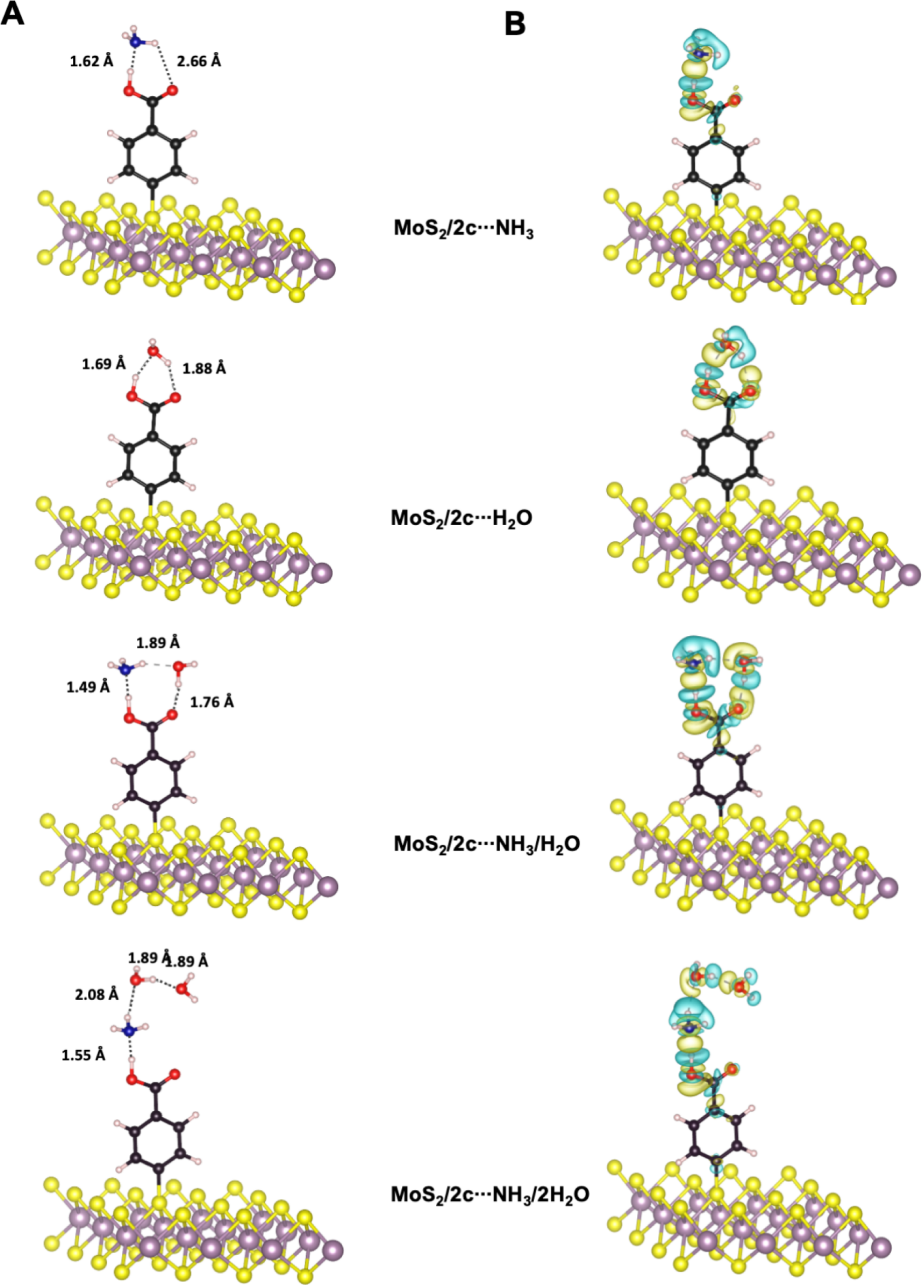
A) Optimized geometries of **MoS**_**2**_**/2c** interacting with NH_3_, H_2_O, and NH_3_ in the presence of H_2_O or 2 H_2_O. Arrows indicate atoms involved in HB. B) Plots depicting the differences in charge density are provided to highlight the areas of charge increase (yellow) and regions of charge decrease (blue). The value of the isosurface plots was 1.5 × 10^–3^ e/Bohr.^2^.

Furthermore, we computed the interaction energy of ammonia in the presence of residual amounts of water molecules present in human breath or atmospheric humidity (Figure 5A).^43,44^ The relaxed structure of **MoS**_**2**_**/2c** in the presence of one NH_3_ molecule and one H_2_O molecule showed that carboxylic acid preferentially interacted with NH_3_, whereas H_2_O interacted with the N–H group of ammonia. The interaction energy obtained was 2.5-fold higher than that obtained using ammonia alone. Furthermore, similar results were obtained for **MoS**_**2**_**/2c** interacting with one NH_3_ molecule and two H_2_O molecules, with interaction energy of −1.04 eV. These results are in contrast to those obtained for ammonia and water molecules when they were tested individually and confirmed the high selectivity of the surface to ammonia.

#### Differences of Charge Density

Calculation of the charge density differences of the functionalized MoS_2_ in the presence of NH_3_ and H_2_O target molecules revealed local variations in electron density in the vicinity of the interaction zone. As shown in Figure 5B, the blue (charge density loss) and yellow (charge density gain) areas around the atoms provided valuable insights into the local changes and differences in electron density induced by the presence of specific atoms in the interaction zone. We noticed the presence of a blue lobe around the acidic hydrogen from the carboxylic acid and charge accumulation (yellow lobe) in the intermediate distance between hydrogen and nitrogen from ammonia. In addition, the latter loses charge density due to its electron–lone pair. Similar conclusions were obtained by examining the interactions between CO_2_H and H_2_O.

In addition, in the case of **MoS**_**2**_**/2c** interacting with one NH_3_ molecule and one H_2_O molecule or one NH_3_ molecule and two H_2_O molecules, one can see from the charge density difference that the molecules are bound together via hydrogen bonds. This can be observed from the accumulation of charge density lobes (yellow lobes) between O and H from the acidic group and N from NH_3_, N–H from NH_3_, O from H_2_O, O–H from H_2_O, and O from O=C. Charge-depletion zones are located around the hydrogen bond acceptors. A similar network of HBs can be seen, involving the HB donors and acceptors from CO_2_H, NH_3_, and two H_2_O molecules.

#### Partial Charges

**Figure 6:**
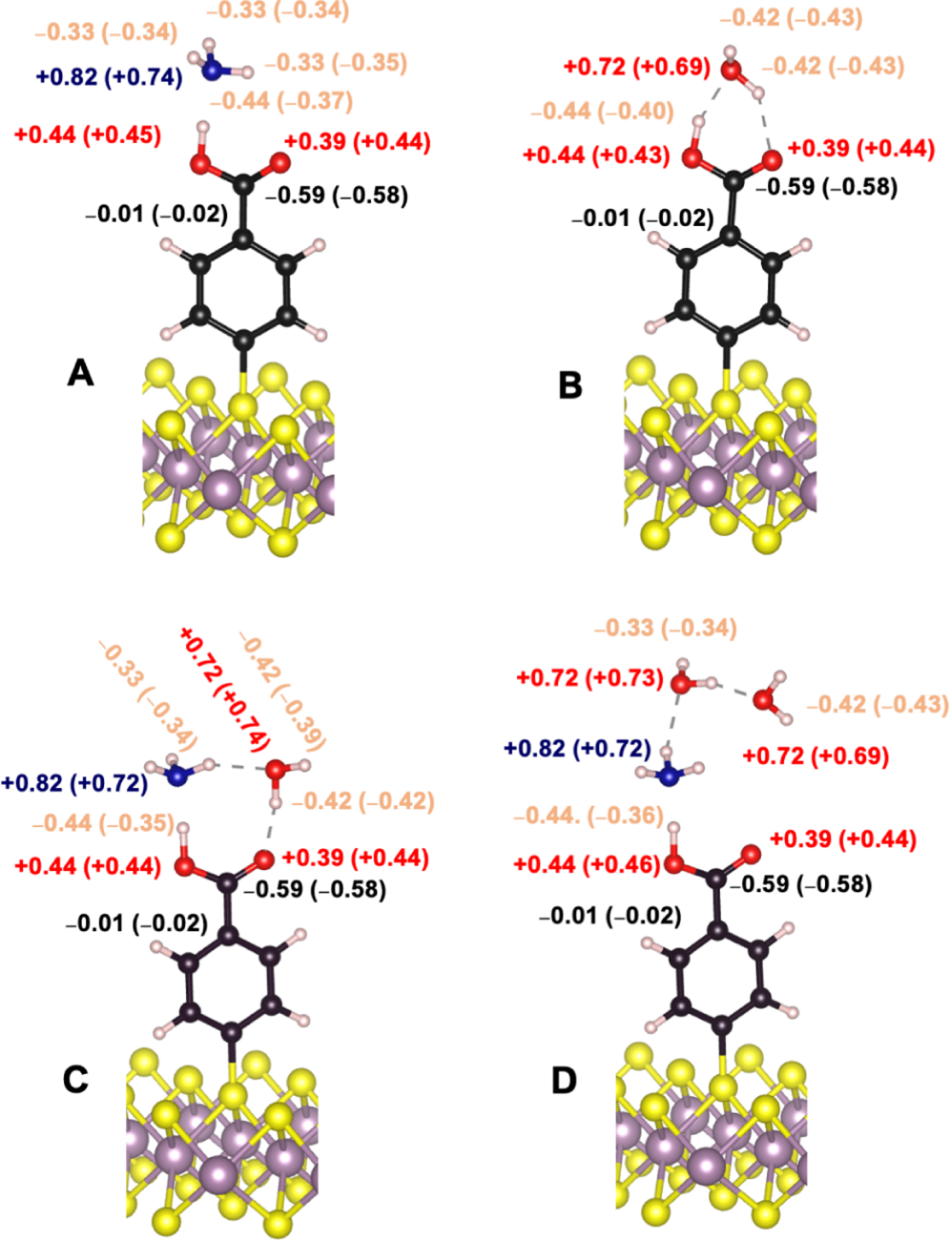
Partial computed charges before and after interaction with (A) NH_3_, (B) H_2_O, (C) NH_3_/H_2_O, and (D) NH_3_/2H_2_O.

The interaction of **MoS**_**2**_**/2c** with NH_3_, H_2_O, NH_3_/H_2_O, and NH_3_/2H_2_O provides fertile ground for exploring Löwdin partial charges. This analysis probes the distribution of electron charges within this crucial interface. The Löwdin partial charges offer a measure of the electron density around atoms, providing insights into the electrostatic interactions between the surface components and detected molecules (Figure 6). The presence of the CO_2_H acid group, which is capable of forming hydrogen bonds, and its interaction with NH_3_ and H_2_O suggest variations in the electronic distribution.

Overall, one can notice that initial charge of the carbon adjacent to the carboxylic acid and the carbon of the carboxylic acid did not change much (−0.01 e) after the interaction with the ammonia, the water or the water and ammonia, denoting the weak effect of the interaction on the distant carbon atoms. The oxygen atoms of carboxylic acids behave differently. Indeed, the O of the O–H group is less sensitive to the interaction since its charge varied by 0.01 to 0.02 e, while the O from C=O group varied by 0.02 e. The most noticeable charge variation occurred with nitrogen from the NH_3_ molecule because its charge dropped by 0.08–0.10 *e*. Water molecules are less sensitive to the interaction because their charges in (B), (C), and (D) varied only by 0.01 to 0.03 *e*. The hydrogen from the O–H group changed more drastically by 0.04 to 0.09 *e* because it was directly involved in hydrogen bonding with ammonia or water molecules.

#### Recovery Times

The characterization of the recovery times in the context of the detection of ammonia and water molecules with **MoS**_**2**_**/2c** offers crucial insights into the dynamics of molecular interactions at the surface (Table 2). These recovery times, measured at different temperatures, offer significant insights into the durability of the bonds formed between the modified surface and the detected molecules. The recovery time refers to the duration necessary for a sensor to revert to 90% of its initial baseline signal after removal of the target gas.^45^

At room temperature (298 K), ammonia showed a recovery time longer than that obtained water molecules, but both values of τ were less than one second (≤0.31 s). At a higher temperature (373 K), both recovery times decreased significantly and were shorter than 1 ms. In the presence of water molecules, the overall interaction energies increased significantly, resulting in longer recovery times at 298 and 373 K. Considering only the interaction occurring between the ammonia and the surface, the calculated durations varied from ∼109 to ∼7882 s at 298 K and from ∼0.2 to ∼5.0 s at 373 K. Using two water molecules, the interaction energy is weaker because of the dense HB network that ammonia formed with H2O molecules. The resulting recovery time calculated at 298 K is only 4.8 s. These values are more realistic since they considered the humidity of the atmosphere or the human breath.

## Discussion

Recently, experimental evidence has demonstrated the functionalization of MoS_2_ nanosheets with diazonium salts.^3,10,46,47^ However, the underlying mechanism of this reaction and its impact on the physicochemical properties of modified materials remains unclear. This research used solid-state DFT to investigate the physical and chemical adsorption of several representative diazonium salts with diverse functional groups positioned para to the diazonium group. Our aim was to understand how these substitutions affected the physicochemical characteristics of the modified materials.

Our findings indicate that diazonium salt cations exhibit poor adsorption on the MoS_2_ surface (with adsorption energies *E*_ads_ < 0.4 eV) when the nitrogen group faces Mo and sulfur atoms. In contrast, the aryl cations generated from the decomposition of diazonium cations react with the surface to form C–S covalent bond, resulting in a significantly higher energy release (*E*_rxn_ > 1.6 eV). Notably, aryl cations containing electron-donating groups exhibited enhanced adsorption compared to the unsubstituted cations and to others with electron-withdrawing groups in the following order: −NH_2_ > −OMe > −H > −CHO > −CO_2_H > −NO_2_ (Table 1). This underscores the facilitation of functionalization, as observed experimentally.^3,10,46,47^ Additionally, the analysis of the band structures, total DOS, and PDOS confirms successful functionalization, leading to local perturbations in the band structure and DOS, thereby influencing the conductivity and optical properties of the functionalized surface.

Of the six modified MoS_2_ surfaces studied, the carboxylic-group-modified surface exhibited the highest interaction with ammonia, water, carbon dioxide, sulfur dioxide, and hydrogen disulfide, which was attributed to the formation of hydrogen bonds with the lone pairs of these molecules. In particular, the interaction energies with ammonia and water molecules were notably high, suggesting strong selectivity of the surface toward these molecules. Further analysis revealed the preference of the surface to form hydrogen bonds with ammonia, whereas water molecules interacted with the N–H group of NH_3_. These results were supported by calculations of the charge density differences, Löwdin partial charges, and recovery times at various temperatures. Notably, the presence of one NH_3_ molecule and one H_2_O molecule or one NH_3_ molecule and two H_2_O molecules results in prolonged recovery times ranging from several hours to thousands of hours, which can be adjusted using laser irradiation to achieve acceptable recovery times ranging from few minutes to approximately 2 h.

Compared to other recent works (Table 3), our findings showed that the interaction with ammonia is more prominent that those reported for MoS_2_/MoO_3_.^48^ The interaction energy values are comparable to those obtained for SO_2_ sensing using Rh-MoS_2_,^49^ but much lower than those obtained for SO_2_ and H_2_S using Ni-MoS_2_.^50^ This underscores the adaptability of the chemical and physical properties of MOS_2_ as a substrate for the detection of various gases.

**Table 3:** Comparison with the Literature in Terms of Efficiency, Selectivity, and Stability of the Sensors at Room Temperature

materials	target gas/Interaction energy (eV)	interferents	response time (s)	stability	ref
MoS_2_/MoO_3_	NH_3_/–0.34	H_2_S, HCHO, EtOH, MeOH, NO	5.9 × 10^–7^	high	(48)
MoS_2_/SnO_2_	NH_3_	CH_4_, H_2_, CO, H_2_S, NO_2_	1.6	high	(49)
Rh-MoS_2_	SO_2_/–0.85	-	234.8	low	(51)
NiO-MoS_2_	H_2_S/–1.32	EtOH, HCHO, H_3_C(O)CH_3_, C_6_H_6_	-	high	(52)
	SO_2_/–1.38			high	
MoS_2_/Co_3_O_4_	NH_3_	EtOH, HCHO, H_3_C(O)CH_3_, C_6_H_6_	105	high	(53)
Pd/MoS_2_	NH_3_	-	1800	high	(54)
	NO_2_				
MoS_2_/WS_2_	NH_3_	EtOH, MeOH, HCHO, H_3_C(O)CH_3_	80	high	(55)
MoS_2_/PhCO_2_H	NH_3_/–0.83	NH_3_	108.7	high	this work
	NH_3_/–0.75	H_2_O	7881.9	high	

## Conclusion

Using a computational approach based on solid-state DFT, we investigated the functionalization of MoS_2_ with diazonium salts to address the limitations of selectivity. Our findings indicate that chemical reactions between sulfur atoms and organic diazonium cations are preferred over physical adsorption. The initial optical properties were preserved when the bandgap energy varied slightly. This study specifically focused on ammonia detection, revealing that substituted MoS_2_ responded to the target analyte with varying interaction energies. Notably, MoS_2_ functionalized with a phenylcarboxylic acid group exhibited the most favorable interaction energy, demonstrating its potential for selective ammonia sensing. Additionally, the nanomaterials exhibited good selectivity when exposed to other gases, such as carbon dioxide, sulfur dioxide, and hydrogen sulfide, as evidenced by the lower interaction energies. This selectivity was further confirmed by calculating the partial charges and recovery times. Our work provides a theoretical background for researchers looking for suitable modifying agents to achieve selectivity toward ammonia using modified MoS_2_ gas sensors.

## Data Availability

The data underlying this study are available in the published article and its Supporting Information.

## Supporting Information Available

Screenshots of the optimized geometries for the different diazonium cations adsorbed on MoS_2_, relaxed geometries for the ammonia interacting with the different aromatic-functionalized MoS_2_, relaxed geometries for CO_2_, SO_2_, H_2_S, H_2_O, 2NH_3_, NH_3_/H_2_O, and NH_3_/2H_2_O interacting with the carboxylphenyl-functionalized MoS_2_ and the band structure of the pristine MoS_2_ (PDF)
