# Supplementary material for: Surface Tailoring of MoS2 Nanosheets with Substituted Aromatic Diazonium Salts for Gas Sensing: A DFT Study
Source: ACS Omega. 2024 Aug 23;9(36):37953–64. doi: 10.1021/acsomega.4c04506 (PMC11391560; doi:10.1021/acsomega.4c04506)
Supplement: Supplementary file 1 — ao4c04506_si_001.pdf [file ao4c04506_si_001.pdf]

Surface Tailoring of MoS_2 Nanosheets with Substituted Aromatic Diazonium Salts for Gas Sensing – a DFT Study

Rabiaa Hajlaoui,[1,2] Sabrine Baachaoui,[2] Sami Ben Aoun,*[3] Said Ridène,*[1] Noureddine Raouafi*[2]

[1]Advanced Materials and Quantum Phenomena Laboratory, Department of Physics, Faculty of Sciences of Tunis, Tunis El Manar University, 2092 Tunis, Tunisia.
[2]Analytical Chemistry and Electrochemistry Lab (LR99ES15), Department of Chemistry, Faculty of Sciences, University of Tunis El Manar, 2092 Tunis, Tunisia
[3] Department of Chemistry, Faculty of Science, Taibah University, P.O Box 30002, Al-Madinah Al-Munawwarah, Saudi Arabia.
*Corresponding Authors: Noureddine Raouafi(noureddine.raouafi@fst.utm.tn); Said Ridène (said.ridene@fst.rnu.tn) and Sami Ben Aoun (sbenaoun@taibahu.edu.sa)

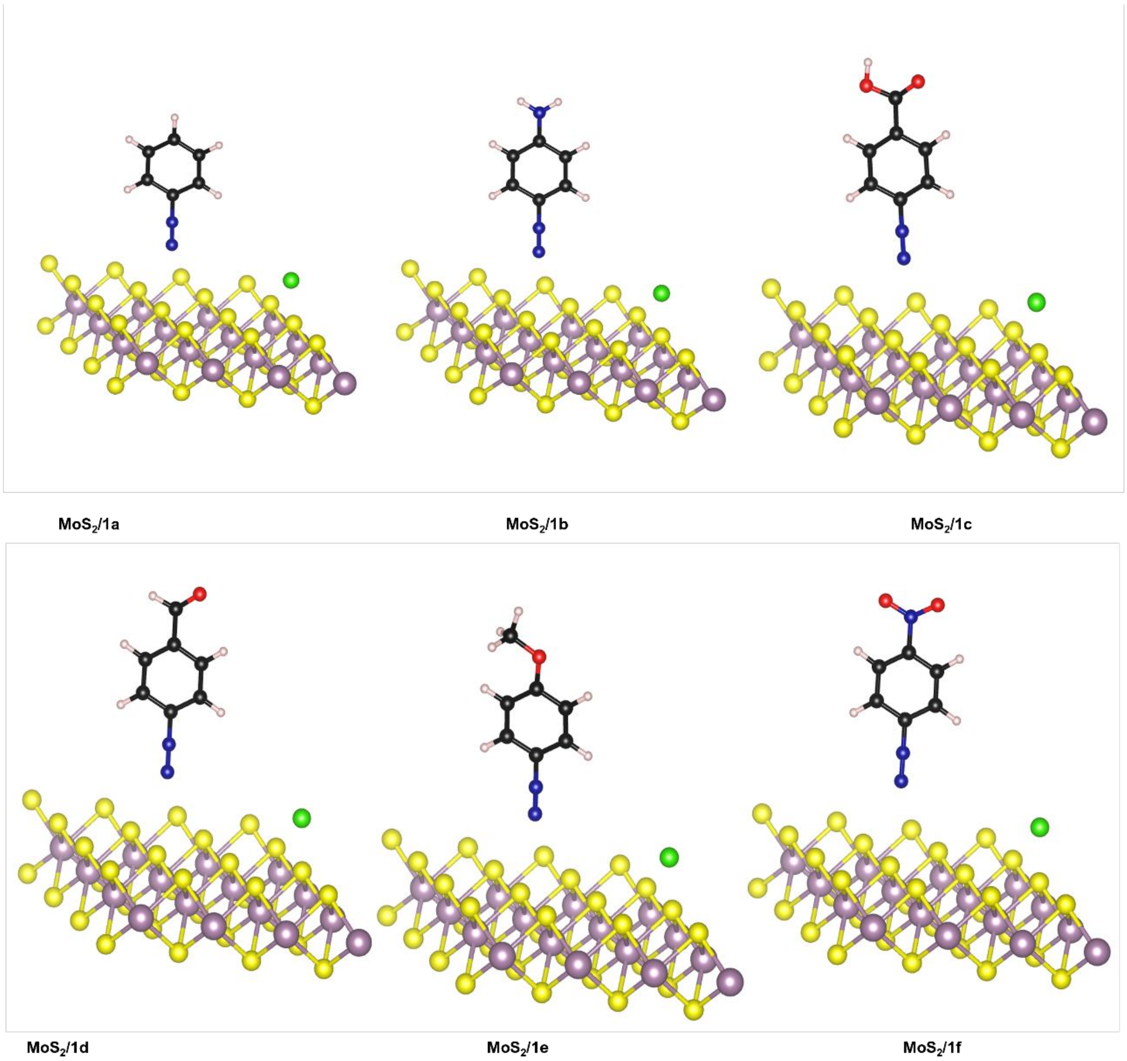

Figure S1: Different geometries optimized at top^{Mo} positions by physical adsorption of diazonium salts on the MoS_2 surface.

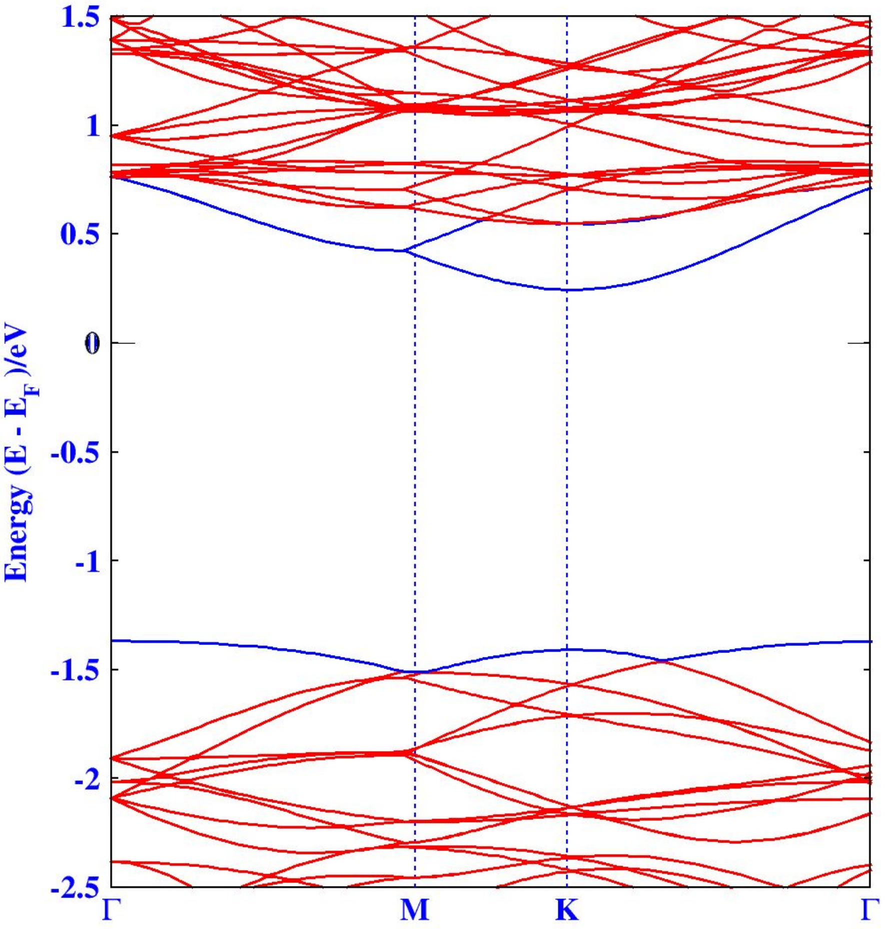

Figure S2: Band structure of pristine MoS_2.

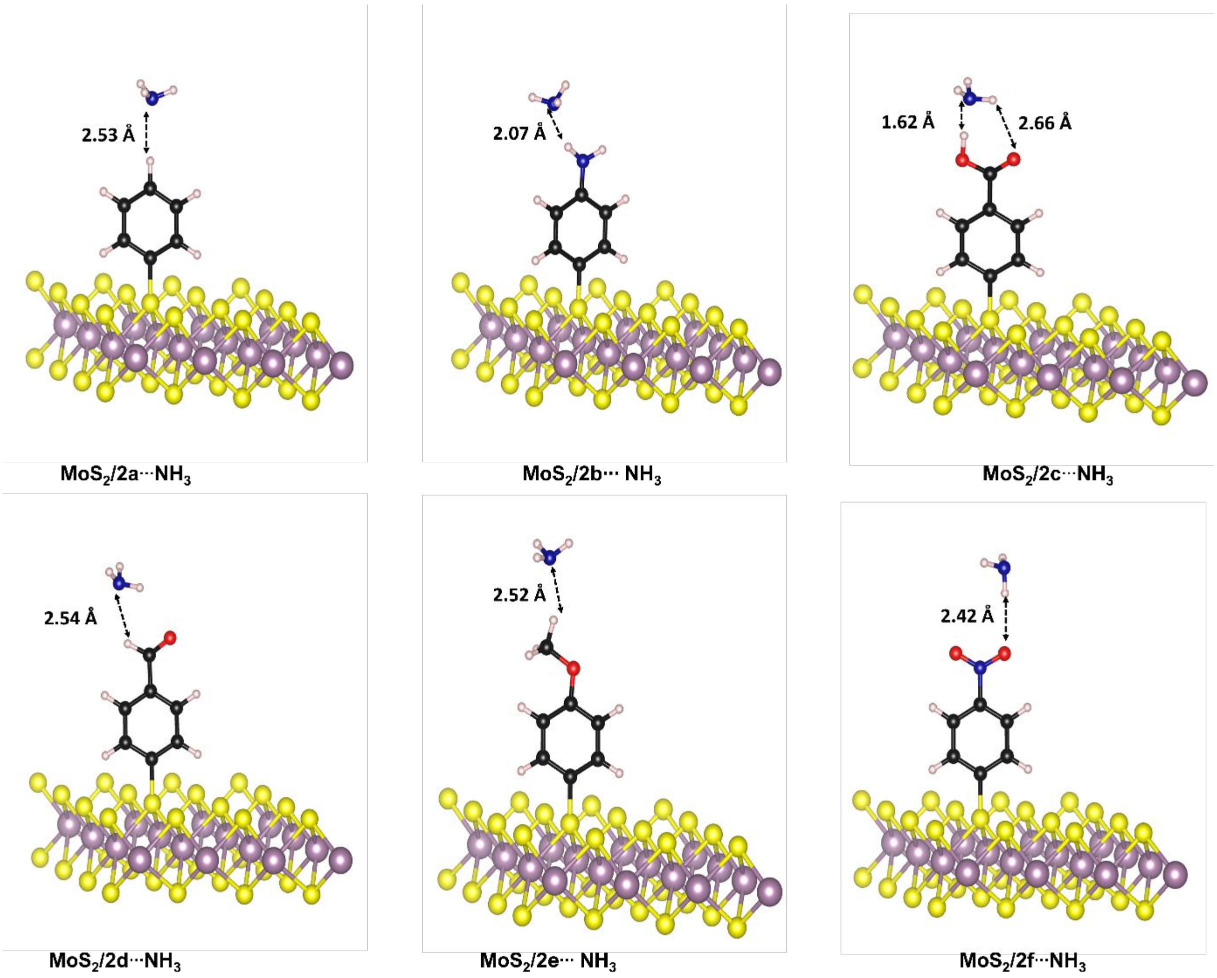

Figure S3: Optimized geometries of different modified MoS_2 substrates interacting with ammonia. Arrows indicating the lengths of hydrogen bonding established between the ammonia and the modified MoS_2.

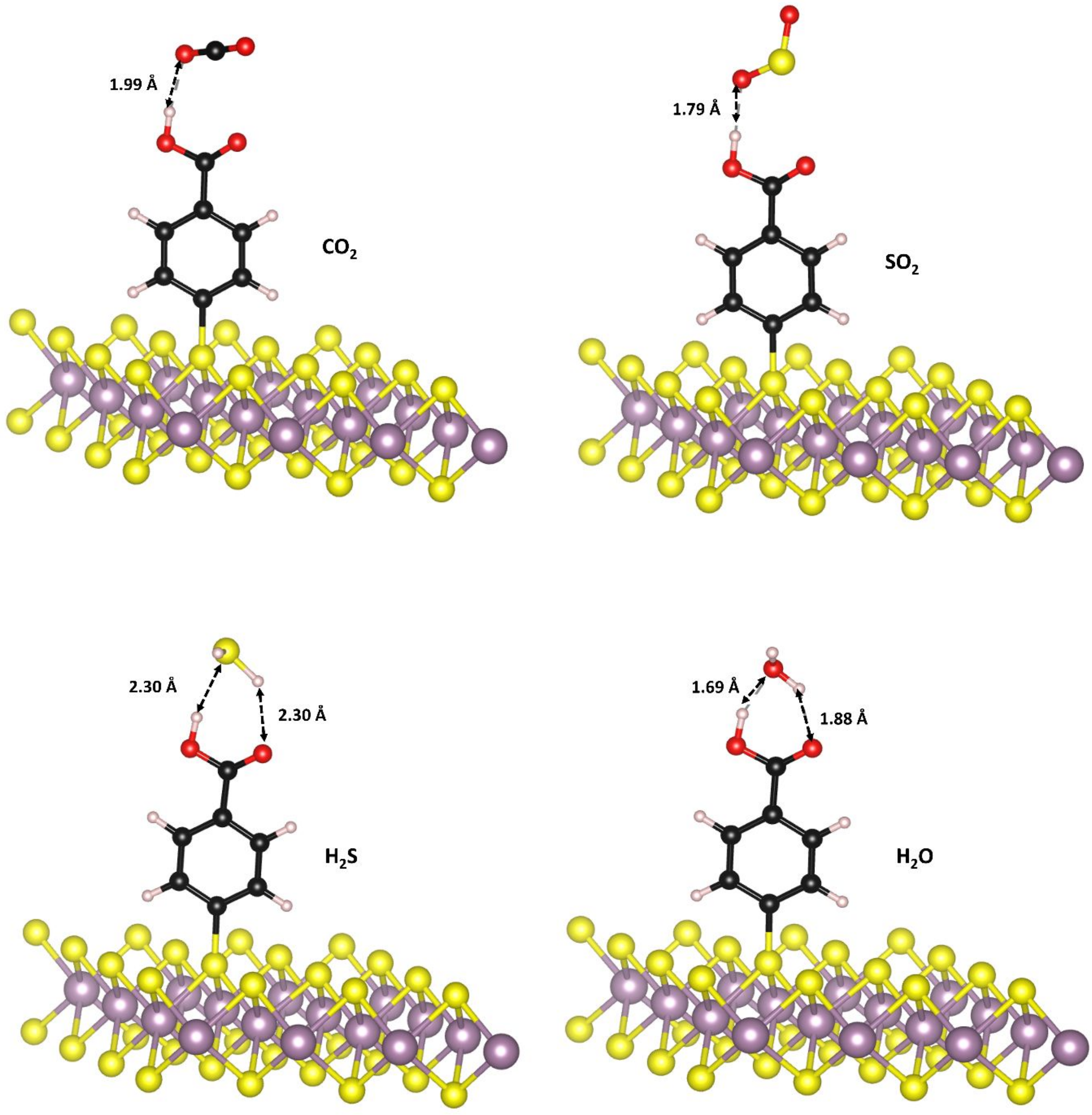

Figure S4: Optimized geometries of **MoS_2/2c** interacting with CO_2, SO_2, H_2S and H_2O molecules.

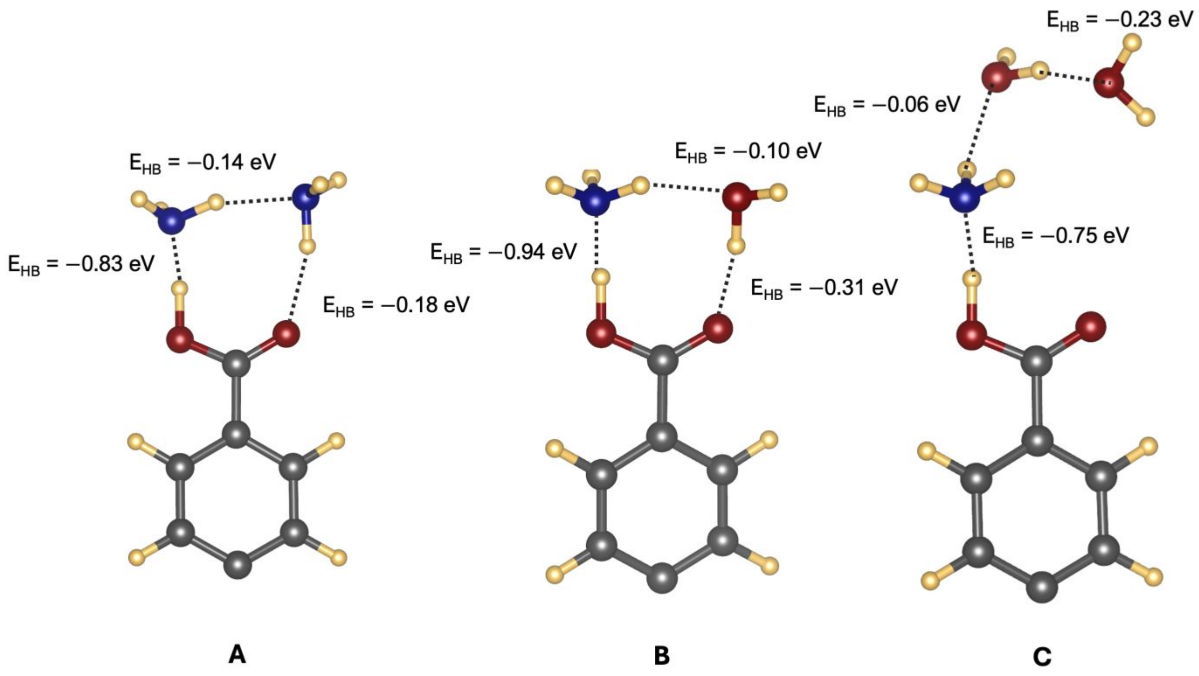

Figure S5: Optimized geometries of MoS_2/2c interacting with 2 NH_3 (A), one NH_3 and one H_2O (B) and one NH_3 and 2 H_2O and the corresponding HB energies.
